# Challenges and innovations in resistance, remediation, and regulation: key findings from the spring 2025 ASM Theobald Smith Society meeting

**DOI:** 10.1128/msphere.00374-25

**Published:** 2025-08-04

**Authors:** Lily Lumkong, Brian Nyiro, Sammi Russo, Isabella Westervelt, Raymond F. Sullivan, Jeffrey M. Boyd, Valerie J. Carabetta, Srujana S. Yadavalli, Jennifer S. Sun

**Affiliations:** 1 Global Health Institute, Rutgers University 5970, New Brunswick, New Jersey, USA; 2 New Jersey Medical School, Rutgers University 67206 , Newark, New Jersey, USA; 3 Department of Biochemistry and Microbiology, Rutgers University 574031, New Brunswick, New Jersey, USA; 4 Department of Biology, William Paterson University, Wayne, New Jersey, USA; 5 Department of Marine and Coastal Sciences, Rutgers University, New Brunswick, New Jersey, USA; 6 Department of Biomedical Sciences, Cooper Medical School of Rowan University 363994, Camden, New Jersey, USA; 7 Department of Genetics, Rutgers University 198473, Piscataway, New Jersey, USA; 8 Waksman Institute of Microbiology, Rutgers University 57675, Piscataway, New Jersey, USA; University of Michigan, Ann Arbor, Michigan, USA

**Keywords:** branch meeting, microbiology, environmental biology, infectious diseases, clinical microbiology, applied microbiology, cellular biology, molecular biology

## Abstract

The annual spring meeting for the Theobald Smith Society was held in May 2025 on the campus of Rutgers University—New Brunswick, as part of a 2-day celebration of microbiology discovery in New Jersey. A total of 140 branch members from across New Jersey attended the meeting, composed of undergraduate, graduate, and postdoctoral trainees, faculty members, and government and industry professionals. This report highlights the breadth and diversity of research conducted by members of the American Society for Microbiology in the Theobald Smith Society and celebrates their groundbreaking discoveries.

## MEETING SUMMARY INFORMATION

The annual spring meeting for the Theobald Smith Society: New Jersey Branch of the American Society for Microbiology (ASM) was held in May 2025 at Trayes Hall in the Douglass Student Center at Rutgers, The State University of New Jersey, in New Brunswick, NJ. The branch includes members from all of New Jersey. The majority of the 140 attendees were trainees (14.62% undergraduate students, 38.46% graduate students, and 15.38% postdoctoral researchers), joined by faculty members and government and industry professionals (Table 1). Regional institutions were well represented (Table 2). The morning keynote, “The spread of multidrug-resistant *Klebsiella pneumoniae*: unraveling a plasmid epidemic,” was delivered by Dr. Barry Kreiswirth, a member of the Center for Discovery and Innovation at Hackensack Meridian Health. The 2025 Waksman awardee, Dr. Donna E. Fennell, delivered the lecture “Above and below: harnessing microbial activity for environmental remediation.” Jason Yang was presented with the 2025 Young Investigator Award. The afternoon keynote, “Addressing the possibility of a histone-like code in bacteria,” was delivered by Dr. Valerie Carabetta, associate professor of biomedical sciences at Cooper Medical School of Rowan University. A total of 60 posters were presented from 12 regional institutions, including Arcadia University, the Center for Discovery and Innovation—Hackensack Meridian Health, Cooper Medical School of Rowan University, Monmouth University, Montclair State University, Princeton University, Robert Wood Johnson Medical School, Rutgers New Jersey Medical School, Rutgers University—Camden, Rutgers University—New Brunswick, Rutgers University—Newark, and William Paterson University. Six trainees were selected to present 10-minute lectures describing their ongoing research endeavors. The meeting concluded with a poster research award presentation and forward-thinking statements in anticipation of ASM Microbe 2025 and the fall 2025 Theobald Smith Society meeting.

**TABLE 1:** Career stage of symposium attendees

Career stage	Count	Percentage
Graduate students	54	39%
Faculty	34	24%
Postdoctoral researcher	22	16%
Undergraduate students	21	15%
Industry	5	4%
Visiting scientists	2	1%
ASM staff	2	1%
Total	140	

**TABLE 2:** Affiliations of symposium attendees

Affiliation	Count	Percentage
ASM	2	1%
Arcadia University	2	1%
Center for Discovery and Innovation	5	4%
Cooper Medical School of Rowan University	6	4%
Industry	5	4%
Mercer County Community College	1	1%
Monmouth University	3	2%
Montclair State University	10	7%
New Jersey Institute of Technology	1	1%
Princeton University	6	4%
Robert Wood Johnson Medical School	1	1%
Rutgers New Jersey Medical School	6	4%
Rutgers School of Dental Medicine	2	1%
Rutgers University—Camden	8	6%
Rutgers University—New Brunswick	68	49%
Rutgers University—Newark	5	4%
Seton Hall University	2	1%
William Paterson University	7	5%
Total	140	

## OPENING REMARKS

In her final address as President of the Theobald Smith Society, Dr. Jennifer S. Sun welcomed over 140 attendees, the largest group in recent memory, and celebrated the growth of the society. She emphasized the value of the ASM community, encouraging membership and participation in its programs, such as ASM Microbe and the ASM Connect platform. Dr. Sun also highlighted the achievements of three Peggy Cotter Travel Award recipients and called for continued volunteer engagement.

Dr. Liisa Veerus, the U.S. Young Ambassador for ASM in New Jersey, spoke about the many benefits of ASM membership for early-career scientists, including mentorship, international student ambassador roles, and access to career and research awards. She also shared her local outreach efforts and invited ideas for future microbiology-related events.

Finally, Adrianna Borgia, managing editor for ASM Journals, provided an overview of ASM’s 15 journals and their inclusive editorial mission. She announced the launch of new initiatives, including the open-access “subscribe to open” model, and encouraged audience participation as authors, reviewers, or editors.

## KEYNOTE ADDRESS: THE PLASMID EPIDEMIC

The morning keynote address at the symposium was delivered by Dr. Barry Kreiswirth, a member of the Center for Discovery and Innovation at Hackensack Meridian Health and a longtime leader in the field of antimicrobial resistance. Known for his foundational work on *Staphylococcus aureus*, *Mycobacterium tuberculosis* (Mtb), and *K. pneumoniae*, Dr. Kreiswirth returned to the Theobald Smith Society, where he was the 2015 Waksman Honorary Lecturer, with a compelling and data-driven talk on the overlooked public health crisis of multidrug-resistant *K. pneumoniae* (MDR-Kp) in the community.

While most attention has historically focused on hospital-acquired carbapenem-resistant *K. pneumoniae* (CRKP), Kreiswirth’s team partnered with Quest Diagnostics to focus on outpatient infections. They sequenced over 2,000 extended-spectrum beta-lactamase-positive *K. pneumoniae* isolates collected from across the U.S., primarily from elderly women with urinary tract infections. Strikingly, 82% of these cases came from outside hospitals. Despite a diverse population of strains—over 120 sequence types—the vast majority (92%) carried the *CTX-M-15* gene on conjugative IncF plasmids. These plasmids often encode resistance to multiple antibiotic classes and harbor traits for environmental survival, including heat and metal resistance.

Dr. Kreiswirth described this as a “plasmid-driven epidemic,” in contrast to the clonal spread seen with earlier CRKP strains. The mobility of *CTX-M-15*, enabled by both plasmid conjugation and transposon-mediated transfer, has allowed it to infiltrate a broad genetic landscape. Long-read sequencing revealed the structure of these hybrid plasmids, and CRISPR-based plasmid curing experiments confirmed their direct role in both antibiotic resistance and environmental persistence.

Of particular concern were the clinical implications: over 75% of isolates were resistant to commonly prescribed oral antibiotics, including fluoroquinolones, nitrofurantoin, and Bactrim. With oral therapies dwindling, even uncomplicated infections may increasingly require intravenous (IV) treatment. Innovative therapies like ceftazidime-avibactam and cefiderocol offer hope, but the urgency of the problem remains.

Kreiswirth concluded by urging the microbiology community to expand surveillance beyond clinical settings. He suggested that antibiotic resistance may be driven more by ecological pressures than clinical use—a hypothesis supported by findings of identical plasmids in wastewater treatment plants. Dr. Kreiswirth’s work reminds us: the war on resistance is not just happening in hospitals, it is happening everywhere.

## 2025 YOUNG INVESTIGATOR AWARD

The Theobald Smith Society awarded Dr. Jason H. Yang, assistant professor and Chancellor Scholar at Rutgers New Jersey Medical School, the 14th Young Investigator Award, in recognition of his exceptional scientific accomplishments and leadership. Dr. Yang’s interdisciplinary career began with dual undergraduate degrees in biomedical engineering and electrical engineering from Johns Hopkins University, followed by a Ph.D. in biomedical engineering from the University of Virginia, where he was named Outstanding Graduate Student. His postdoctoral work at the Massachusetts Institute of Technology (MIT) and the Broad Institute propelled him to the forefront of medical engineering, infectious diseases, and systems biology.

Currently, Dr. Yang leads the Systems and Synthetic Biology Lab at Rutgers, tackling critical health issues like antimicrobial resistance, tuberculosis (TB), and heart failure. His innovative research combines high-throughput experimentation, network modeling, and machine learning to create predictive models that advance precision medicine. His work has earned him prestigious awards, including the NIH K99/R00 Pathway to Independence Award and the Agilent Early Career Professor Award.

During the spring symposium, Dr. Yang inspired attendees with a message of cautious optimism, encouraging civic engagement and persistence in science. Honoring Dr. Yang, the Society recognizes not only his research contributions but also his dedication to mentoring and scientific resilience.

## KEYNOTE ADDRESS: WAKSMAN AWARD LECTURE

Dr. Donna E. Fennell, professor and chair of the Department of Environmental Sciences at Rutgers University—New Brunswick, delivered the 72nd Waksman Honorary Lecture with warmth, humor, and deep scientific insight. Her talk traced a career devoted to harnessing microbial processes to address environmental contamination, from aquifers polluted with industrial solvents to dioxin-laced river sediments and even the microbial life of the atmosphere.

Dr. Fennell opened by introducing the concept of the “malenclave,” a term coined in 1965 by Henry Legrand to describe pockets of groundwater rendered unusable by pollution. These zones, common across the United States and even beneath Rutgers’ Cook and Douglass campuses, often harbor dense chlorinated solvents like trichloroethylene (TCE) and perchloroethylene that sink into aquifers, resist removal, and migrate unpredictably. Initially thought to be non-biodegradable, these chemicals were historically treated using pump-and-treat systems and air stripping technologies. Ironically, the microbial fouling of these systems hinted at a new possibility that microbes might not only survive in these toxic environments but could help clean them up.

Through decades of pioneering research, Fennell and her collaborators helped demonstrate that obligate organohalide-respiring bacteria, such as *Dehalococcoides mccartyi* strain 195, could completely dechlorinate TCE to ethene, a relatively benign end product. This finding laid the groundwork for modern bioremediation strategies, including commercial products that deliver both microbes and the electron donors they need to thrive underground. Her research emphasized how careful tuning of organic substrates, such as lactate or butyrate, can stimulate dechlorination without promoting methane production, a crucial balance for safety and efficacy.

The lecture then shifted to river sediments, focusing on the highly contaminated lower Passaic River in Newark, NJ, one of the most polluted waterways in the world, historically impacted by Agent Orange production. Even after years in cold storage, sediment samples from the river revealed microbial communities capable of degrading not only chlorinated solvents but also extremely toxic dioxins and furans. Fennell’s lab has identified and characterized key microbial players, including *Dehalogenimonas* and novel bacteria with organohalide-respiring capabilities. These studies continue through a major Superfund research effort targeting similarly affected rivers in Michigan.

In a more recent and unexpected research direction, Dr. Fennell described her team’s investigations into microbial activity in the atmosphere. Collaborating with Gedi Mainelis and Lee Kerkhof, her lab built rotating air microcosms from 55-gallon drums to study whether airborne microbes remain metabolically active. Using stable isotope probing, they showed that some methanotrophs could incorporate methane-derived carbon into their DNA, providing the first direct evidence of active microbial metabolism in the atmosphere. These findings suggest that airborne bacteria may play a previously unrecognized role in global methane cycling and climate dynamics.

Throughout the lecture, Dr. Fennell highlighted novel strategies for enhancing bioremediation, including the use of slow-release, human-safe electron donors like emulsified vegetable oil, polylactate, and chitin. These tools are helping to transform environmental remediation into a safer, more targeted, and more sustainable practice. She also reflected on the influence of her mentors and the importance of scientific flexibility in response to changing environmental and societal needs.

With more than 100 publications and $7.8 million in research funding as principal investigator (PI) or co-PI, Dr. Fennell is widely recognized as a leader in environmental microbiology and engineering. Her lecture underscored the powerful role of microorganisms in solving real-world environmental challenges—beneath our feet, in our rivers, and even in the air we breathe. As she reminded the audience, the microbes are always working. We just have to learn how to work with them.

## KEYNOTE ADDRESS: BACTERIAL HISTONE-LIKE CODE

The afternoon keynote by Dr. Valerie Carabetta, associate professor of biomedical sciences at Cooper Medical School of Rowan University, delivered a compelling challenge to longstanding assumptions in microbial gene regulation. Her central question was “Could bacteria possess a regulatory system analogous to the eukaryotic histone code?” Drawing on a combination of genetic, biochemical, and structural approaches, Dr. Carabetta presented strong evidence that such a system may exist in the form of reversible post-translational modifications to chromatin-like proteins in bacteria.

Dr. Carabetta’s team focuses on HBsu, a nucleoid-associated protein in *Bacillus subtilis* and a member of the conserved HU family. HBsu plays a key role in bacterial chromosome organization, and her team has identified seven lysine residues on the protein that undergo acetylation. Using site-directed mutagenesis to create acetylation-mimic (K→Q) and deacetylation-mimic (K→R) variants, her group showed that six of the seven sites significantly influence DNA compaction. Mutants mimicking acetylation consistently exhibited relaxed nucleoids, while deacetylation mimics promoted hyper-compaction, indicating that lysine acetylation modulates HBsu-DNA interactions. Structural models supported these observations: for example, K37, whose mutation had no impact on nucleoid structure, faces away from DNA, suggesting that modification at this site does not influence chromatin architecture.

This regulation extends beyond DNA packing. Dr. Carabetta highlighted the role of HBsu acetylation in *B. subtilis* sporulation, a developmental program initiated under nutrient stress. One site in particular, K41, emerged as a key regulatory node. Acetylation-mimic mutants at K41 (K41Q) exhibited precocious expression of sporulation genes such as *sigF* and *spoIIQ*, leading to earlier spore formation. However, these spores were less robust, suggesting that untimely activation of the sporulation pathway compromises spore quality. In contrast, other acetylation-deficient mutants showed delayed or reduced sporulation, pointing to a site-specific regulatory network governing developmental timing. The data support a model in which HBsu acetylation fine-tunes sporulation dynamics, reminiscent of histone modifications that regulate developmental transitions in eukaryotes.

Dr. Carabetta also examined how HBsu modifications influence antibiotic stress responses. Under kanamycin treatment, wild-type *B. subtilis* cells condensed their nucleoids, a likely protective mechanism. In contrast, mutants mimicking acetylation at K3 and K75 failed to compact their DNA, and K41Q mutants showed enlarged nucleoids. These structural changes correlated with altered persistence phenotypes: acetylation-mimic mutants exhibited delayed recovery after antibiotic removal. Together, the results suggest that site-specific acetylation and deacetylation coordinate chromatin remodeling during stress, regulating both entry into and exit from the persister state. In this model, antibiotic stress triggers deacetylation at sites like K3 and K75 to promote compaction and dormancy, followed by re-acetylation at K41 to initiate regrowth.

To support the notion of a bacterial “code,” Dr. Carabetta’s team has been investigating the enzymes responsible for placing and removing these modifications. They have identified four acetyltransferases—YfmK, YdgE, YdhI, and YokD—that act on HBsu, with YdgE showing a strong preference for modifying K41. The NAD^+^-dependent deacetylase SrtN is currently under investigation as a potential regulator of HBsu deacetylation, and preliminary *in vitro* data suggest that it is capable of removing acetyl groups from histone-like peptides. These enzymes appear to be differentially regulated under nutrient limitation and antibiotic exposure, adding another layer of dynamic control.

Dr. Carabetta’s work reframes our understanding of bacterial gene regulation. Far from being limited to simple transcription factor-mediated responses, her findings suggest that bacteria deploy reversible post-translational modifications to chromatin proteins to control major physiological transitions. Her ongoing efforts to decode these modifications and identify the enzymes that regulate them point toward a bacterial “epigenetic” system with profound implications for microbial physiology, synthetic biology, and antimicrobial drug development.

## PRESENTATION THEMES

The oral and poster presentations were distributed across the three scientific units defined by ASM, reflecting the breadth of research represented at the meeting: ASM Health (29.41%), ASM Applied and Environmental Microbiology (30.88%), and ASM Mechanism Discovery (39.71%).

## ASM HEALTH

The ASM Health theme highlighted innovative strategies for addressing infectious diseases, with a strong emphasis on antimicrobial resistance and host-pathogen interactions. Several poster presentations showcased novel diagnostics and therapeutic targets, underscoring the importance of mechanistic insight in guiding translational advances.

On the diagnostics front, Lily Lumkong (Rutgers University—New Brunswick) presented a rapid reverse-transcription recombinase polymerase amplification (RT-RPA) assay for *La Crosse virus* detection, capable of identifying viral RNA within 20 minutes. Beyond its clinical utility, the assay enabled dissection of age-dependent neuroinvasion and viral clearance in a murine model, offering a valuable tool for studying viral pathogenesis alongside its diagnostic potential.

A number of studies focused on antifungal resistance, particularly mechanisms driving echinocandin resistance in *Candida glabrata*. Zubayeda Uddin (William Paterson University) demonstrated that the cell wall integrity pathway, specifically the kinase SLT2, is essential for FKS2-mediated resistance. Intriguingly, disrupting SLT2 reversed resistance in *fks2* mutants but not *fks1*, suggesting that SLT2-dependent signaling differentially supports resistance depending on the FKS isoform. A related poster introduced NAT1, a small molecule that targets SLT2 itself, providing a pharmacological avenue to sensitize *fks2* mutants to echinocandins. These findings underscore the therapeutic potential of targeting stress-response pathways to combat fungal drug resistance.

Additional insights into potential antimicrobial targets emerged from studies of bacterial stress adaptation. Brian Gavarrete (William Paterson University) showed that deletion of *mrp* and *pha* gene clusters in *Marinobacter adhaerens*, involved in multi-resistance, pH adaptation, and ion transport, disrupted pH homeostasis and sodium/potassium balance, identifying these antiporters as possible intervention points in marine or extremophile pathogens.

Geordan Stukey (Rutgers University—New Brunswick) identified sertraline, a widely used antidepressant, as an inhibitor of yeast Pah1 and human lipin 1 phosphatidate phosphatases. This unexpected activity raises the possibility of repurposing sertraline as a modulator of lipid metabolism in fungal or even human disease contexts.

Host-microbiome interactions rounded out the session. Sherry Chen (Rutgers University—New Brunswick) explored interspecies communication between skin commensals *Cutibacterium acnes* and *Staphylococcus epidermidis*, using spent media assays to uncover antagonistic or synergistic interactions that may contribute to skin health or disease.

Finally, Arkajyoti Dutta (Rutgers University—New Brunswick) uncovered a particularly urgent therapeutic target with the identification of CC10501, a small molecule that binds the P-stalk pocket of the ricin A subunit and inhibits the catalytic activity of both ricin and shiga toxins. With no approved therapeutics for these potent biotoxins, this work represents a significant step toward viable countermeasures.

Together, these studies illustrate how an integrated approach—from enzymology and genetics to microbiome interactions and small molecule screening—is driving progress in combating infectious diseases and antimicrobial resistance.

## ASM MECHANISM DISCOVERY

The ASM Mechanism Discovery theme featured a wide range of research across disciplines, unified by a focus on fundamental mechanisms of microbial physiology, drug resistance, host-pathogen interaction, and model development for disease pathogenesis. Collectively, these presentations advanced our understanding of how microbes adapt to host environments, resist treatment, and shape infection dynamics.

Drug resistance and pathogenesis in *Mycobacterium* were major themes. Ahri Han (Rutgers New Jersey Medical School) presented work on host-pathogen interactions between Mtb and macrophages, aiming to identify targets for host-directed therapies. Treating TB remains a long, toxic process with an increasing risk of drug resistance. Han focused on PE/PPE proteins, which constitute about 10% of the Mtb genome yet remain understudied. They showed that PPE-15 harbors a SH3-like domain allowing interaction with NWASP, a key actin assembly protein, potentially altering macrophage motility. Follow-up studies will investigate infection outcomes using knockout models. Related poster presentations explored other PE/PPE family members involved in autophagy regulation and type I interferon production during infection.

Julianna Proietto (Hackensack Meridian Health) developed a new model for chronic *Mycobacterium abscessus* infection using agar bead-embedded bacteria in immunocompetent mice (1). This approach overcomes the limitations of existing models, which are rapidly cleared by innate immunity. The embedded bacteria established infection for at least 3 months, creating a chronic infection platform more representative of human disease and useful for testing new drug regimens against this increasingly drug-resistant pathogen.

Additional mechanistic insights were shared by Gustavo Rios-Delgado (Rutgers University—New Brunswick), who studied how *S. aureus* adapts to iron limitation during infection. His work identified the small RNA IsrR as a Fur-regulated repressor of aconitase (*acnA*), helping explain why iron-starved *S. aureus* mutants have impaired tricarboxylic acid (TCA) cycle activity and growth on amino acids. IsrR mutants restored *acnA* levels and metabolic function, highlighting IsrR’s broader role in nutrient sensing and pathogenesis (2).

Drug delivery mechanisms were addressed by Joshua Chamberlain (Rutgers University—Camden), who investigated how adding sphingolipids alters the behavior of lipid nanoparticles. These systems, crucial for RNA delivery and vaccine development, depend on lipid composition for stability and efficacy. By examining zeta potential and membrane dynamics, the team showed that sphingolipid addition enhanced *in vivo* stability and circulation time, potentially improving the pharmacokinetics of fragile therapeutics.

Basic science studies on bacterial biosynthesis and regulation further expanded the session. Angela Zhu (Princeton University), winner of the second-place poster award, identified a new class of ribosomally synthesized and post-translationally modified peptides called cysimiditides. These peptides, found across Actinomycetota, require zinc-bound cysteine motifs to convert aspartic acid to aspartimide, a previously undescribed metal-dependent modification. Their signature mass shift and unique biosynthesis could serve as a platform for discovering novel antimicrobial compounds.

Tanisha Dhakephalkar (Rutgers University—Camden), winner of the third-place poster award, investigated sphingolipid biosynthesis in *Caulobacter crescentus*. This bacterium uniquely survives without lipopolysaccharide by synthesizing ceramide polyphosphoglycerate (CPG2). She identified and characterized the enzyme CpgD, a phosphoglycerate cytidylyltransferase that synthesizes CDP-glycerate from CTP and 3-phosphoglycerate—a key precursor in CPG2 biosynthesis. Enzyme kinetics and metal dependence confirmed its function, filling a longstanding gap in understanding bacterial sphingolipid pathways.

Mechanistic enzymology also featured prominently. Karina Mejias, Citlaly Hernandez, Crystal Montero, and Stella Obuba from Dr. Nina Goodey’s lab (Montclair State University) dissected the activity of indole-3-glycerol phosphate synthase (IGPS), an enzyme essential for tryptophan biosynthesis in both *Mycobacterium tuberculosis* and *Thermotoga maritima*. They identified proton transfer as the rate-limiting step in MtIGPS catalysis, mapped the functional consequence of N189D and N189L mutations on MtIGPS activity, and explored the thermostability and catalytic performance of TmIGPS, showing that the E47D mutation reduced activity but improved stability, valuable for biotechnological applications. Together, these studies refine our understanding of IGPS function and highlight its potential as a drug target.

Taken together, these oral and poster presentations illustrated the depth and breadth of work being done to interrogate microbial mechanisms at the molecular level—from pathogenesis and immune evasion to novel model development, enzyme function, and therapeutic design.

## ASM APPLIED AND ENVIRONMENTAL MICROBIOLOGY

The ASM Applied and Environmental Microbiology theme explored microbial signaling, parasitic enzymology, and environmental microbiomes, highlighting both fundamental discoveries and translational potential.

Francis J. Santoriello (Princeton University) presented his work on quorum sensing and phage-host interactions, focusing on the VP882 phage system. VP882 uses a phage-encoded quorum-sensing mechanism to regulate the lysis-lysogeny decision, mediated through the production of Qtip in response to host-derived signals. Santoriello showed that the phage interprets both quorum sensing and environmental cues upon host entry to decide between lytic replication and lysogeny. His future work aims to synthetically reprogram this decision-making process by engineering phages to respond to custom-designed inducers, potentially enabling fine-tuned control of phage behavior for therapeutic or synthetic biology applications.

From Montclair State University, David Otu-Aboagye and Salma Kwarteng focused on developing improved treatments for lymphatic filariasis by targeting *Wuchereria bancrofti* dihydrofolate reductase (DHFR), an essential enzyme for nucleotide synthesis. Current treatments like methotrexate are limited by toxicity due to their effects on human DHFR. The team is working to identify parasite-specific DHFR inhibitors through compound screening and protein crystallization, using methotrexate-like compounds TSD compounds as leads. Their aim is to develop more selective, less toxic alternatives for treating this debilitating parasitic infection.

Environmental microbiomes and bioremediation were also a highlight. Sage M. Phelps and Paul X. Santarsiero (Monmouth University), recipients of the first-place poster award, investigated microbial communities in the pitchers of *Sarracenia purpurea*, a carnivorous plant native to the nutrient-poor Pine Barrens of New Jersey. These plants depend on symbiotic microbial communities to digest captured insects and supply nutrients. The team sampled seven field sites across seasons, using both culture-based methods and marker-gene sequencing (16S rRNA and ITS) to identify bacteria such as *Chromobacterium vaccinii* and *Rahnella aquatilis*. Their ongoing work aims to determine how environmental conditions, plant health, and seasonal variation shape these micro-ecosystems, offering broader insight into community assembly and succession in specialized plant-microbe systems.

These studies underscore the diversity and depth of applied microbial research conducted by Theobald Smith Society constituents, highlighting the value of integrating molecular insight with ecological context and therapeutic application.

## CONCLUSIONS

The spring 2025 symposium of the Theobald Smith Society highlighted many pressing areas of concern to researchers, particularly antimicrobial resistance mechanisms, environmental remediation strategies, and the molecular basis of bacterial adaptation and survival. The research reflects the growing interdisciplinary approach required to gain substantial insights into the microbial landscape. For meeting details, including all abstracts, go to https://www.njmicrobe.org/spring-2025-symposium. All the oral presentations presented at the meeting can be viewed at https://www.youtube.com/@TheobaldSmithSociety. A more detailed review of the symposium can be downloaded at https://drive.google.com/file/d/1np3n7yOK8Xh9opzHKlKmHXDOnEU7c9UL/view?usp=sharing.
